# Policy processes underpinning universal health insurance in Vietnam

**DOI:** 10.3402/gha.v7.24928

**Published:** 2014-09-26

**Authors:** Bui T. T. Ha, Scott Frizen, Le M. Thi, Doan T. T. Duong, Duong M. Duc

**Affiliations:** 1 Department of Reproductive Health, Hanoi School of Public Health, Hanoi, Vietnam; 2 Department of Arts and Sciences, New York University Shanghai, Shanghai, China

**Keywords:** universal health insurance, universal health coverage, policy process, Vietnam

## Abstract

**Background:**

In almost 30 years since economic reforms or ‘renovation’ (*Doimoi*) were launched, Vietnam has achieved remarkably good health results, in many cases matching those in much higher income countries. This study explores the contribution made by Universal Health Insurance (UHI) policies, focusing on the past 15 years. We conducted a mixed method study to describe and assess the policy process relating to health insurance, from agenda setting through implementation and evaluation.

**Design:**

The qualitative research methods implemented in this study were 30 in-depth interviews, 4 focus group discussions, expert consultancy, and 420 secondary data review. The data were analyzed by NVivo 7.0.

**Results:**

Health insurance in Vietnam was introduced in 1992 and has been elaborated over a 20-year time frame. These processes relate to moving from a contingent to a gradually expanded target population, expanding the scope of the benefit package, and reducing the financial contribution from the insured. The target groups expanded to include 66.8% of the population by 2012. We characterized the policy process relating to UHI as incremental with a learning-by-doing approach, with an emphasis on increasing coverage rather than ensuring a basic service package and financial protection. There was limited involvement of civil society organizations and users in all policy processes. Intertwined political economy factors influenced the policy processes.

**Conclusions:**

Incremental policy processes, characterized by a learning-by-doing approach, is appropriate for countries attempting to introduce new health institutions, such as health insurance in Vietnam. Vietnam should continue to mobilize resources in sustainable and viable ways to support the target groups. The country should also adopt a multi-pronged approach to achieving universal access to health services, beyond health insurance.

Vietnam is a socialist country under the leadership of a single party (Communist Party of Vietnam – CPV). In 1986, Vietnam started the reform ‘*Doimoi*’ from planned economy toward a socialist oriented market economy with the aim of unleashing the strength, dynamism, and creativity of its people (1). The policy was fantastically successful, with GDP per capita in real terms increasing from 130 USD (1990) to 1911 USD in 2013 (2). Economic growth has contributed to improving the living standards and the health status of the population; it has, at the same time, facilitated increasing government investment in health care (3).

In the past 20 years, Vietnam has achieved good health outcomes, in many respects with results similar to those of high middle-income countries. Vietnam is among the countries that are on track to achieve different Millennium Development Goals, including reduction in child mortality rates (4, 5). However, there are increasing inequities of health among regions (6).

Recently, the concept of universal health coverage (UHC) was widely advocated by the WHO and other stakeholders. UHC is a system in which everyone in a society can avail the health care services they need without incurring financial hardship (7). There are three main core factors to consider in UHC: who is covered, which service is covered, and the level of financial contribution (8). The UHC required the comprehensive approach with health service delivery, health financing, and political economy and policy processes (9). Although UHC has become a major goal for health reform in many countries, its achievement typically requires an incremental and systematic policy approach.

Although the policies in Vietnam are not consistent with a comprehensive interpretation of UHC, the government started financing curative care through health insurance (HI) in 1992 (10). Over 20 years, four key policy documents on HI were in effect, that is, Decree 299/1992, Decree 58/1998, Decree 63/2005, and Law on HI (2008) and Decree 62/2009 guiding Law implementation. In 2013, the government published its roadmap to achieve universal health insurance (UHI) coverage levels of 70% by 2015 and 80% by 2020 (11).

Although UHI offers a powerful aspirational goal for Vietnam, there are many challenges associated with adopting, achieving, and sustaining it; balancing various political and economic influences is among the most important challenge. In 2012, the coverage of HI in Vietnam was in practice only 66.8% (10), and many shortcomings have been identified in low coverage, lack of basic service package, and insufficient health financing. There is a growing demand to understand the policy levels that can influence the attainment of UHI objectives. This study aims to enhance the HI policy-making processes in Vietnam. This study attempts to describe the UHI policy processes and influential political economy factors influencing UHC in Vietnam.

## Methods

The qualitative research methods (in-depth interview, focus group discussion (FGD), expert consultancy, and secondary data review) were applied in this study. The fieldwork was conducted in two provinces in 2013 (Hanoi and Haiduong) with four FGDs and 30 in-depth interviews. The purposive sampling (snowball technique) was used to identify the key informants (Table 1).

**Table 1:** Key informants

No.	Participants	Working agencies	No.	Methods
1	Policy designers/policy-makers	MOH, VSS	5	5 In-depth interviews
2	Politicians	Government Office, Central Party	2	2 In-depth interviews
3	International partner	WHO	1	1 In-depth interview
4	Administrators	Provincial Health Department and Social Insurance	6	6 In-depth interviews
5	Implementers	Hospitals (public and private), clinics, health insurance agencies	23	2 FGDs and 10 in-depth interviews
6	Users	Commune health centers, provincial and district hospitals, private hospitals	17	2 FGDs and 6 in-depth interviews
	Total		54	4 FGDs and 30 in-depth interviews

A review of 420 documents related to the main policies on HI was conducted, including articles, policies, training materials, media, research, and projects. The research team had sought all identified full-text studies in Vietnamese and English. The research reports could be unpublished, published, or written on HI and UHI in Vietnam from 1990 to 2013. Studies with no data or without published full-text were excluded. The research used electronic databases (PopMed, Ebscohost, Science-Direct) and printed reports of organizations (government or non-government), institutions, or research centers. The research team at the Hanoi School of Public Health, supported by a World Bank consultant, carried out the analysis.

All the interviews/FGDs were conducted in Vietnamese. All interviews/FGDs were recorded and transcribed. Content analysis was applied. The data were analyzed with the support of NVivo 7.0 (12). The conceptual framework, drawing on Walt and Gilson's policy triangle on content, context, and actors in health policy processes underpinned the analysis (13). Analysis was done in Vietnamese and the report was written in English.

Discussions with, and validation by, all research partners and key policy-makers in Vietnam were used to ensure the reliability and validity of findings. Ethical approval was obtained from the Institutional Review Board of Hanoi School of Public Health.

## Results

**Fig. 1:**
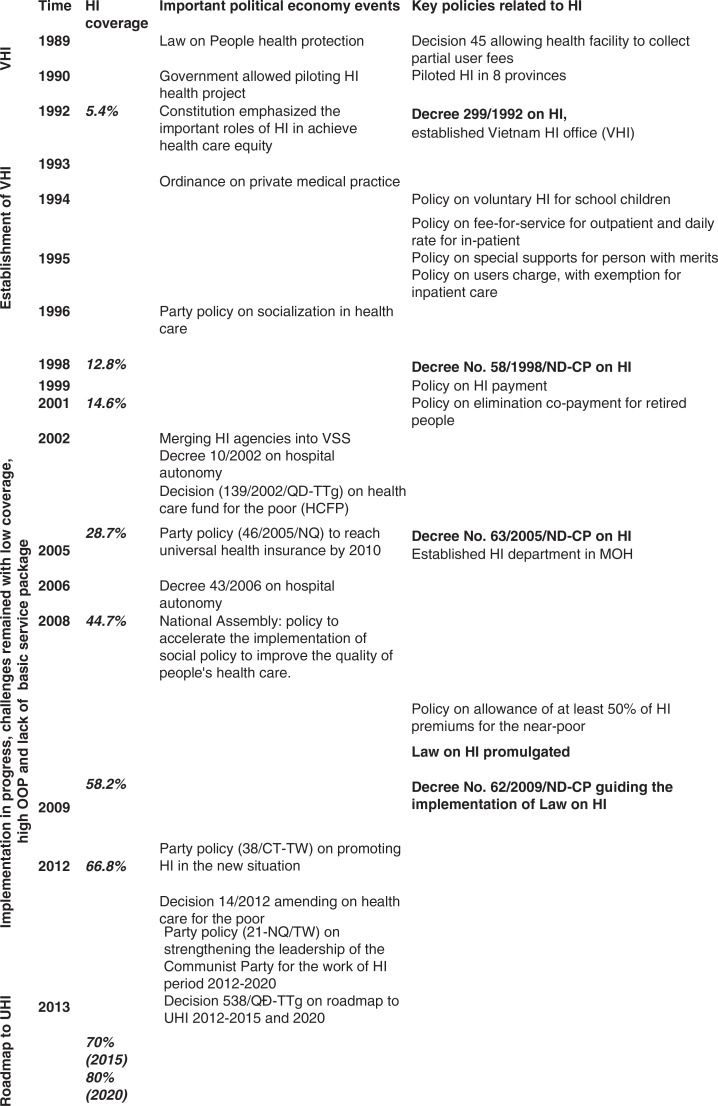
Timeline of key health insurance processes in Vietnam.

The results of the HI policy process are described in two main parts: the first HI policy, and the following policies. The first part presented the initiation and piloting of the HI policy. The following part described HI policy adoption, expansion, and roadmap to UHI policy (Fig. 1).

### 1990s: Agenda setting and development of the first HI policy in the context of non-experience and non-precedence

#### Context

In Vietnam, before *Doimoi* 1986, the government provided and funded all health care activities, including preventive and curative care. Vietnam experienced socioeconomic crisis because of the collapse of the socialist system in the Soviet Union and Eastern Europe in the late 1980s, which resulted in the sudden cut of foreign aid which in turn affected the government's ability to fund all health care activities. There was deterioration in the health sector in the early 1990s and decline in funding for all public services (14). After *Doimoi*, Vietnam adopted a market economy policy while retaining socialist features in its government structure. The government promulgated policy on partial hospital fees to sustain the health care system in 1989 and the Ordinance on Private Medical and Pharmaceutical Practices in 1993. The Circular 14/1995/TTLB on partial user fees was introduced in 1994 (15). This pushed the health sector to find additional financial sources to run the health system.

At the same time, some key policies were developed such as the Law on People's health protection (1989) and the Constitution of 1992. The right of all people to health care was identified in the above policies providing the legal basis for integrating UHI with national support and resources.

#### Policy development/actors

The CPV had a strong leadership and stewardship toward HI in Vietnam, especially the Commission of Central Propaganda. There was much concern about the poor financial resources of the health system. Dr. Tran Khac Long from the Health Department at Central Propaganda was sent to the Ministry of Health (MOH) to respond to the health financial project. Dr. Long had reviewed the documents (Bismarck model in health care). The documents were from his training materials in East Germany. According to Dr. Long's proposal, HI is one of the options to organize health financing system in reaching UHC to many developed countries where the set of basic health care services is accessible to all, irrespective of income or social status (16).

This HI should be for people and by the people. This was very new – nobody understood about HI. Now, government must pay for HI, employer must pay for the employees and employees must pay. So, this was a very new concept. (HN-VSS-developer)

Between 1989 and 1992, voluntary non-commercial HI was piloted by Dr. Tran Khac Long from MOH and health managers in Quang Tri province and eight districts in the context of the public resources met only 30% of the health care needs and out-of-pocket (OOP) was high at 70%. The project was labeled ‘The State and People working together’. Children and elderly were the main target groups. The program demonstrated that poor local people could share the costs with the health care sector by contributing products such as rice and sugarcane as premium payment for purchasing equipment and medicines.

Lessons learned from the pilot project were used in the development of the first draft Ordinance on HI in 1991 led by the MOH. However, the draft was not approved by the National Assembly (NA) because of concerns over its financial feasibility. Later, Dr. Long and the MOH revised this content within Decree level (the level of Decree is lower than Ordinance, and does not have to be submitted to NA for approval) and submitted it to the office of the Prime Minister (PM). The PM supported the Decree and immediately approved the HI Decree in 1992, just 3 months after submission. The HI head office, which was established right after the Decree approval, played a key role in implementing the HI policy.

#### Content of Decree 299/1992

The HI was started compulsory for civil servants and pensioners as the easiest way to apply the policy. The premium for HI accounted for 3% of salary. The service package included both outpatient and inpatient services.

#### Policy implementation/actor involvement

Fund deficit was observed in 20 provinces during initial implementation of Decree 299/1992, especially in poor provinces and those with high number of pensioners. High expenditure on drugs and a high length of stay for inpatients was reported. These problems were partly resolved by MOH later, when policies on fees for services (Circular 14/1995) and ceiling fees (Inter-ministerial Circular No. 17/1997) were passed.

During the implementation of the Decree 299/1992, much support came from several international partners such as Intensive World Health Organization Cooperation, Swedish International Development Agency, Information System Securities Awareness, and International Labor Organization on strengthening capacity of MOH on HI delivery programs. The support was mainly related to improving human resource capacity such as training on HI, seminars on planning, financial management, and other related activities.

### From 1998 to present: UHI policy process: incremental processes with learning-by-doing approach

#### Context

In order to advance the market economy and increase the resources used for the public sector, the CPV and the government implemented several policies on social mobilization, decentralization, and autonomy. These policies allow the public health sector to raise the necessary resources to provide better equipment and infrastructure, and increase service availability in the health care system. However, these also contributed to increasing inequity of UHI coverage and distributing resources across the different pools.

In this time, the growth of private sectors was gradually increasing. The private beds accounted for 3% of total beds and 1.4% of total autonomous hospitals by 2012 (15).

The financial sources for health care are from different channels: general taxation, social HI, and out-of-pocket payment. There are two major public financial sources that supply funding to health care, namely the state budget allocated directly to service providers, through the MOH and the flow from the social HI fund. Of the total government budget, 93% is for recurrent spending and only 6.85% is meant for spending for development, including equipment and construction. Recently, the government has increased state budget on health from 5.22% (2005) to 8.7% (2010) and 9.4% (2012) (15). Economic growth, while instrumental in supporting the expansion of coverage, also has significant influence on the institutional arrangements for HI delivery. Commitment of the Vietnam government to ‘poor reduction’ during 2000–2010 also played a key role in the UHI coverage.

#### Content

The Vietnam government gradually expanded the service package of HI. The pathway that the Vietnam government applied since 1998 was one of providing HI for the whole population with a larger basic benefit package, funded through a range of financing mechanisms, with the poor and the ethnic people exempted from payment. The service package was gradually expanded (17). There was no limitation in the package covered by HI on drugs, tests, diagnostic imaging, or surgery and medical procedures (17, 18).

#### Policy process/actors involvement

The development of HI policies was identified as a response to the major shortcomings encountered during the implementation of previous policies, including low coverage, lack of basic service package, HI fund deficits, and lack of effective financing mechanisms. The government through the Health Care Fund for the Poor provided the premium payments for the poor and near poor (Decision No. 139/2002), which contributed sharply to increasing coverage. Before 1995, the coverage of HI was low. The coverage of HI reached 5.94% by 1994 (19). By 2012, HI coverage reached 66.8% (20).

By 2005, observing the rapid increasing HI coverage, the CPV set goals of UHI by 2010 (Resolution 46/2005). The roles of HI were recognized as important tools to ensure health care equity as well as important financial sources for health service delivery. The role of the private sector in the health care system was also recognized from 2005.

The lesson was HI could not be successful if it was implemented in a small scale. HI should be implemented in the whole nation. (HN-MOH-policymaker) There should be UHI. This is the mean to ensure the equity, efficiency and development. (HN-politician)

MOH played a significant role in the policy process. The department of HI (which was established in 2005 on the basis of separation from Vietnam health insurance office-VHI) was responsible for developing the policy/law. Several actors have been involved in the development of these policies, including the related ministries, government offices, provincial health departments, and international development partners (mainly World Bank, WHO, and UNICEF). Consultation took place through roundtable meetings and workshops. Users and implementers at the grassroots level were not invited for the debates. Problems from the previous implementation were discussed to be resolved in the agenda setting of the next policy process.

The debates and comments during the development process centered on several issues, including 1) complicated procedures for HI patients; 2) the need for reimbursement in several categories, such as infertility; 3) the need for having a basic, predetermined, benefit package; 4) concerns about the low quality of services at lower levels of the health system and at private facilities; 5) the name of the law; 6) the co-payment rate; and 7) premium rate.

According to key informants, constraints surfaced during the drafting process, including the lack of agreement among and varied perspectives of policy-makers, and the limited time for comments.

The NA members tended to demand lots of benefits, such as that the benefit package not be fixed and the premium reduced, and patients able to go directly to central level facilities to see specialists without being referred by the lower level. But this is not what we mean by ‘social HI’. (HN-international partner)

The department of HI of MOH finalized the draft policy according to comments received, and it was submitted to the government office and the NA for approval. MOH explained to NA members the reasons to revise or not revise the content based on evidence from the facts. The process centered on the development of Law on HI, with 25 versions of draft law before the final bill was approved by the NA on 14/11/2008.

#### Implementation

Funds deficit were evident since the implementation of first policy on HI (Decree 299/1992) and mainly associated with the fact that HI was voluntary, adverse selection problems, the lack of ceiling costs for HI services, the abuse of medical services without co-payment, an ambiguous medical service package, and the increasing costs of medical services.

Fee-for-service was still the predominant payment method. The capitation provider payment mechanism was piloted in four provinces in 2005 with the implementation of Decree 63/2005. Later, this method was expanded to the district level. However, there was much criticism over its inconsistent application among provinces, inappropriate guidelines for capitation payment, and fund deficits at the district level.

It was so hard to for health manager to implement HI policy at district level. Lack of detail guidelines for capitation payment makes us stress. (HD-FGD-manager at district level)

## Discussion

The UHI process in Vietnam has come a long way from its establishment in 1992 to the establishment of a roadmap to get to 70% coverage by 2015 and 80% by 2020. The implementation of UHI is in progress with challenges. The overall policy-making process is incremental with a ‘learning-by-doing’ approach, and reflects the influence of a number of political economy factors.

### Incremental policy processes with learning-by-doing approach

The first policy on HI (Decree 299/1992) was adopted at a time when there was very little policy capacity in the Vietnamese health system with respect to HI. The financial sources from HI were not considered as important and premium rate was set without any detailed financial forecasts. Most policies used some form of evidence, whether in the form of lessons learned from other countries, from pilot project evaluations, or from the results of initial rounds of policy implementation in HI programs, with revisions made to amend shortcomings on an ongoing basis. These small steps paved the way for some major policy changes (21). However, evidence from policy studies or implementation researches was rare. The advantage is that the policy would fit with the low economic development context of Vietnam. However, the disadvantage is non-scientific evidence may limit the radical change in policy content. UHI policies in Vietnam focus more on coverage than they do on the service package and financial protection. As a result, the implementation of UHI is in progress with challenges concerning the lack of essential health services, insufficient financing payment, and fund deficits. These shortcomings must be addressed if Vietnam is to achieve UHC in a comprehensive manner.

The findings clearly showed the processes of discussion, negotiation, contestation, and consensus among different stakeholders in the policy processes. At the central level, the relationship among ministries is horizontal. All ministries are under the stewardship of the NA, government, and CPV in agenda setting. Within each sector, there was a vertical relationship between the central, policy-making level and the implementation level. Overall, these actors, at both the central and the implementation levels, have strong voice and power that could influence the HI policy development and implementation. The voice of other actors, including media, international agencies, and users is still limited because of not being invited to the policy development process. Yet, the policy processes remained, for the most part, closed, with inputs solicited ‘by invitation only’.

### Influence of political economy factors, progressive universalism toward UHI

The HI processes in Vietnam reflect the strong influence of political context factors. The right of all people to health care was identified in a number of policies after *Doimoi* providing the legal basis for national support of UHI. CPV leadership plays a key role in the development of UHI.

There are two pro-poor pathways to achieving UHC within a generation. First, HI would cover basic health package/intervention to achieve convergence, health problems. The second pathway provided a larger benefit package, funded through a range of mechanisms, with the poor exempted from payments (22). Vietnam applied the first approach in the early stage of policy process and applied the second type of progressive universalism in the HI policies from 2005. The advantage of the second approach is that a wide range of health services can be offered, the non-poor are engaged in a prepaid mandatory scheme, and the poor can access health services easier without paying user fees. The situation of Vietnam is similar to that of Rwanda (22). Vietnam is moving to UHI through mandatory insurance and co-payment with exemption for the poor and ethnic people.

Similar to other countries that applied the second type of progressive universalism, Vietnam faced disadvantages (23). It was reported that there was increasing provision of unnecessary services to patients, higher out-of-pocket spending on hospital care, and higher spending per treatment episode, affecting the quality of services and increasing the risk of capitation fund deficit at lower level facilities due to autonomy reformed policies which were first introduced at the district and provincial levels since 2005 (18, 23, 24). These problems were solved via incremental actions such as capitation methods applied at the district and the commune levels, and accepted co-payment for advanced quality services/or treatment at provincial and central levels.

The economic recession in recent years has also led to a slowdown of government financial contributions to the health sector, especially in government bonds for investment in hospitals, and in the limited quality of and accessibility to services. This will affect the desirability and affordability of HI programs in the eyes of both enterprises and individuals, and slower expansion of coverage levels can be expected in the coming years. In summary, it can be seen that the government was looking for market-oriented ‘instruments’ of policy in the new context, while retaining or expanding equity goals.

## Conclusions

The review of the policy processes of UHI over 20 years has shown that Vietnam has attained considerable coherence in the process of developing a comprehensive HI system that contributes to a sustainable and equitable health sector. The results also suggest that policy processes of UHI are mainly taking an incremental, ‘learning-by-doing’ approach with the involvement of a complex array of actors.

The government lays more emphasis on increasing coverage than service package expansion and financial protection. Political economy factors have a significant influence on VHI-related policy processes. In the presence of: 1) decentralization and 2) a prevailing socialist ideology and practice of socialization of health services and autonomy, the disparities both in terms of outcomes and implementation processes have increased. Considerable challenges remain, as achieving high coverage in the informal sector and boosting the voluntary purchase of insurance will not be easy.

This study suggests that an incremental, learning-by-doing approach can be effective and necessary in early stage in the context of low economic development of Vietnam. For the future, Vietnam will need to mobilize resources in sustainable and viable ways to support target group expansion and financial protection, as it makes strides to achieve truly comprehensive HI coverage.

Besides, Vietnam should open opportunity for international actions to UHI process through policy and implementation research. The evidence from research can be used for effective designing and implementing of specific pathways for evolution in the policy for UHI implementation.
